# Longitudinal Cognitive Assessment After CAR-T Cell Immunotherapy: A Prospective Cohort Study

**DOI:** 10.3390/cancers18111803

**Published:** 2026-06-01

**Authors:** Evlampia Strongyli, Anna Papakonstantinou, Christos Demosthenous, Zoi Bousiou, Anna Vardi, Despina Mallouri, Panagiotis Dolgyras, Ioannis Batsis, Paschalis Evangelidis, Ioannis Kyriakou, Marianna Masmanidou, Ioannis Giokaris, Maria Gavriilaki, Asimina Bouinta, Evangelia Yannaki, Damianos Sotiropoulos, Sotirios Papagiannopoulos, Dimitrios Kazis, Vasilios Kimiskidis, Ioanna Sakellari, Eleni Gavriilaki

**Affiliations:** 1 Endothelial Injury Excellence Centre, Bone Marrow Transplant Unit, Hematology Department, G. Papanicolaou Hospital, 57010 Thessaloniki, Greece; evlabia1@gmail.com (E.S.); christosde@msn.com (C.D.); boussiou_z@hotmail.com (Z.B.); anna_vardi@yahoo.com (A.V.); dmallouri@gmail.com (D.M.); panadolg@gmail.com (P.D.); iobats@yahoo.gr (I.B.); mariannareti@gmail.com (M.M.); giokaris.gpapanikolaou@n3.syzefxis.gov.gr (I.G.); alexis.menia@gmail.com (A.B.); dsotiro@otenet.gr (D.S.); ioanamarilena@gmail.com (I.S.); elenicelli@yahoo.gr (E.G.); 2 Faculty of Health Sciences, School of Medicine, Aristotle University of Thessaloniki, 54124 Thessaloniki, Greece; 3 Second Propedeutic Department of Internal Medicine, Hippocration Hospital, Aristotle University of Thessaloniki, 54642 Thessaloniki, Greece; pascevan@auth.gr; 4 1st Department of Neurology, AHEPA University Hospital, Aristotle University of Thessaloniki, 54636 Thessaloniki, Greece; mariagavri6@yahoo.gr (M.G.); kimiskid@auth.gr (V.K.); 5 3rd Department of Neurology, G. Papanicolaou Hospital, Aristotle University of Thessaloniki, 57010 Thessaloniki, Greecedimitrios.kazis@gmail.com (D.K.)

**Keywords:** CAR-T cell therapy, cytokine release syndrome, immune effector cell-associated neurotoxicity syndrome, mini-mental state examination, Montreal cognitive assessment, neurotoxicity

## Abstract

(1) Background: Cognitive dysfunction represents an emerging concern in chimeric antigen receptor T-cell (CAR-T) therapy recipients, yet longitudinal data using simple, clinically applicable tools are lacking. (2) Methods: We conducted a single-center prospective cohort study of consecutive adults with hematologic malignancies treated with commercially available CAR-T cell products between May 2023 and November 2025 at our center. Cognitive function was evaluated with the Montreal Cognitive Assessment (MoCA) and Mini-Mental State Examination (MMSE) at baseline (before the administration of lymphodepleting chemotherapy) (T1), 6 h after infusion (T2), at 3 months (T3), and at 6 months (T4). MoCA scores ≤ 25 and/or MMSE scores ≤ 23 were considered indicative of impaired cognitive function. (3) Results: Thirty-six patients were enrolled in the present study, while cytokine release syndrome occurred in 33/36 patients (91.7%), and immune effector cell-associated neurotoxicity syndrome of any grade occurred in 23/36 (63.9%). At baseline (T1), cognitive impairment was identified in 12/36 patients (33.3%) by MoCA. Following infusion (T2), 11/35 (31.4%) exhibited cognitive impairment, while baseline cognitive impairment and older age were associated with early post-infusion cognitive dysfunction. Across follow-up (T3 and T4), no significant overall changes were observed in MoCA- or MMSE-defined cognitive status or in total test scores. However, abstraction in MoCA and attention/calculation in MMSE showed time-dependent variation. (4) Conclusions: These findings support the use of simple longitudinal cognitive assessment in CAR-T recipients.

## Simple Summary

In this prospective single-center cohort study, we assessed cognitive function in 36 consecutive adult patients with hematologic malignancies receiving chimeric antigen receptor T-cell therapy. Cognitive function was evaluated with the Montreal Cognitive Assessment (MoCA) and Mini-Mental State Examination (MMSE) tests before lymphodepleting chemotherapy, 6 h after infusion, and at 3 and 6 months. Cognitive impairment was common at baseline, particularly when assessed with the Montreal Cognitive Assessment. Early after infusion, a similar proportion of patients exhibited cognitive impairment, whereas older age and pre-existing cognitive deficits were associated with early post-infusion dysfunction. Across follow-up, overall cognitive status and total test scores remained largely stable, with no significant longitudinal decline. However, selected domains, including abstraction and attention/calculation, showed time-dependent variation. These findings indicate that simple longitudinal cognitive testing is feasible in routine practice and may help identify patients at higher risk for early cognitive deficits after treatment.

## 1. Introduction

Over the last few years, chimeric antigen receptor T (CAR-T) cell immunotherapy has revolutionized the treatment landscape for B-cell hematological malignancies, such as B-cell acute lymphoblastic leukemia (B-ALL), relapsed or refractory (R/R) non-Hodgkin lymphomas (NHL), and multiple myeloma (MM). CAR-T cell manufacturing involves isolating and genetically engineering the patient’s T cells to express a specific altered T-cell receptor [1,2]. First-generation CAR-T cells consist of a single-chain fragment extracellular antigen-recognition domain, linked directly to a transmembrane domain, which is then connected to a CD3 intracellular signaling domain. Second-generation CAR-T cells consist of an additional co-stimulatory domain (usually 4-1BB or CD28), and third-generation CAR-T cells combine two signaling domains (typically CD28 and 4-1BB) [3]. Tisagenlecleucel (Tisa-cel), axicabtagene ciloleucel (Axi-cel), lisocabtagene maraleucel (Liso-cel), and brexucabtagene autoleucel (Brexu-cel) are anti-CD19 CAR-T cell products commercially available for the management of patients with B-ALL and specific subtypes of NHL [4,5]. Specifically, Tisa-cel is indicated for the treatment of patients younger than 25 years old with R/R B-ALL [6] and patients with R/R diffuse large B-cell lymphoma (DLBCL) [7]. Moreover, Axi-cel is approved for R/R DLBCL [8]; Brexu-cel for patients with R/R mantle cell lymphoma (MCL); and Liso-cel for R/R DLBCL, primary mediastinal B-cell lymphoma, transformed follicular lymphoma, and MCL [9]. Additionally, idecabtagene vicleucel and ciltacabtagene autoleucel (second-generation CAR-T cells targeting the B-cell maturation antigen [BCMA]) have recently been approved for R/R multiple myeloma (MM), with overall response rates of up to 76% [10] and 97% [11], respectively. Several other CAR-T cell products for R/R MM are under investigation in ongoing clinical trials, including durcabtagene autoleucel (PHE-885), a fully human BCMA-directed CAR-T, with promising results [12].

CAR-T cell therapy offers significant curative potential for patients with B-cell malignancies. Nevertheless, they still may experience significant toxicities, most notably, cytokine release syndrome (CRS), an acute systemic inflammatory syndrome that is characterized by fever, organ dysfunction, and hypotension, and immune effector cell-associated neurotoxicity syndrome (ICANS). Both adverse events are guided by rapid T-cell expansion, resulting in cytokine release [1]. CRS is reported in 37 to 93% [4], whereas neurotoxicity is observed in 50 to 65% of patients receiving CAR-T cell products [13]. Neurotoxicity may manifest with a wide range of neurological symptoms, such as delirium, aphasia, tremors, confusion, ataxia, hallucinations, and seizures [3]. CD28-based CAR-T cells expand faster and tend to cause higher toxicity rates [14], while 4-1BB–based CAR-T cells expand more slowly and are generally associated with lower toxicity rates [3].

CRS and ICANS share pathophysiological similarities with endothelial injury syndromes observed after hematopoietic cell transplantation (HCT), including endothelial dysfunction, the release of inflammatory cytokines, and the development of a procoagulant state [15,16]. Consequently, the Endothelial Activation and Stress Index (EASIX), which was initially developed for the prediction of poor outcomes in allogeneic HCT settings, and its altered version, the modified EASIX, have been validated as predictors of CRS, ICANS, and overall survival in real-world CAR-T cell recipients [17,18]. These patients may also experience several other toxicities, such as hematological toxicities, infections, cognitive dysfunction, and even impaired quality of life [19,20,21].

Management of CRS includes, and is not limited to, supportive measures (intravenous fluids and oxygen supplementation), administration of tocilizumab (blocker of the interleukin [IL]-6 receptor), corticosteroids, anakinra (IL-1 receptor antagonist), and prophylactic antimicrobial therapy, due to the challenging differential diagnosis from infections [22].

Indeed, cognitive impairment represents an emerging issue for CAR-T cell patients. Thus, various diagnostic approaches have been implemented in clinical practice for the evaluation of the CAR-T cell recipients’ cognitive function following infusion [23]. Comprehensive, frequent neurological examinations in patients presenting with neurological symptoms, including ICANS grading, assessment with the immune effector cell-associated encephalopathy score (ICE), and monitoring of neurological symptoms, are widely used in everyday clinical practice. Interestingly, these examinations have been found effective in the identification of cognitive deficits, especially in the first two weeks after CAR-T infusion [24]. During this period, cognitive impairment is considered a manifestation of ICANS, which may be life-threatening [25,26].

Montreal Cognitive Assessment (MoCA) represents an easy and readily available tool, which can assess the following domains: orientation to place and time, short-term memory, language, visuospatial abilities, and higher cerebral functions, such as working memory, attention, and concentration [27]. The MoCA test has been used for the timely detection of cognitive dysfunction in CAR-T cell recipients during a short-term period following infusion (1 month) [28]. Similarly, the Mini-Mental State Examination (MMSE) test is an easy and valuable tool to assess cognitive function promptly in five areas: orientation, language, registration, attention and calculation, and recall after CAR-T cell therapy [29,30]. Both the MMSE and MoCA have a maximum total score of 30 points [31]. MoCA and MMSE are widely used, validated bedside screening tools that can be administered rapidly and repeatedly without specialized equipment. Their simplicity and clinical applicability make them particularly suitable for longitudinal monitoring in patients undergoing complex treatments such as CAR-T cell therapy. However, most of the published studies on cognitive dysfunction following CAR-T cell therapy focus on short-term cognitive deficits, or they use complex tools that cannot be easily used in everyday clinical practice [23].

Given the lack of longitudinal studies using validated tools, we aim to prospectively study neurocognitive effects in adult CAR-T cell therapy recipients with hematological malignancies. By analyzing data at specific time points, we seek to provide insight into the recipient’s cognitive function after CAR-T infusion, improve timely recognition of complications, and ultimately contribute to the optimization of patient care in this quickly developing field.

## 2. Materials and Methods

### 2.1. Study Design, Setting, and Participants

This was a single-center, observational, prospective cohort study that included consecutive adult patients (≥18 years old) with hematological malignancies who received commercially available CAR-T cell therapy according to internationally established protocols [2,32]. Patients treated between May 2023 and November 2025 at our Joint Accreditation Committee International Society for Cell and Gene Therapy–European Society for Blood and Marrow Transplantation (EBMT)-accredited center were considered eligible and were consecutively enrolled after providing written informed consent. Patients with severe neurological or psychiatric disorders that could interfere with cognitive testing, as well as those who declined participation, were excluded from this study. Patient follow-up lasted at least six months after CAR-T cell infusion. Additionally, loss of follow-up during the study period was recorded and reflected in the number of patients available at each evaluation time point.

All patients received lymphodepleting therapy before CAR-T cell infusion, with cyclophosphamide and fludarabine administered according to the respective CAR-T cell product-specific protocol. CAR-T cell products were administered as single infusions based on the respective product-specific prescribing information or investigational study protocol. Neurologists and intensive care specialists contributed to the rigorous monitoring of patients receiving CAR-T cell products in accordance with the guidelines of the EBMT and MD Anderson Cancer Center [2,33]. The American Society for Transplantation and Cellular Therapy grading system was used to diagnose and grade CRS and ICANS [34]. During the first 2 weeks following infusion, patients were monitored daily with electroencephalography (EEG) and ICE assessments for detection of neurotoxicity. Relevant clinical and laboratory data were collected from the patients’ electronic medical records. This study was approved by the Institutional Review Board of George Papanikolaou General Hospital and was conducted in accordance with local legislation and institutional requirements [32].

Cognitive function was assessed using the MMSE and MoCA at four predefined time points: T1 (before the administration of lymphodepleting chemotherapy), T2 (6 h post-infusion), T3 (3 months post-infusion), and T4 (6 months post-infusion). T1 was selected to evaluate baseline cognitive function before lymphodepleting chemotherapy. T2 was selected as an early standardized post-infusion assessment, before later toxicity-related events and therapeutic interventions might further influence cognitive performance. T3 and T4 were selected to capture intermediate and longer-term cognitive outcomes at 3 and 6 months after infusion, respectively. These time points were not chosen based on individual treatment response patterns. Cognitive assessments were performed by a trained registered nurse using standardized versions of the MoCA and MMSE tests, which have been translated and validated for use in the Greek population. Educational level was recorded for all participants, and the official MoCA education correction was applied for patients with ≤12 years of formal education [35]. Briefly, the MoCA assesses visuospatial/executive function, naming, attention, language, abstraction, delayed recall, and orientation, whereas the MMSE evaluates orientation, registration, attention, calculation, recall, and language. Cognitive impairment was defined using commonly used cut-offs. Specifically, MoCA scores ≤ 25 and/or MMSE scores ≤ 23 were considered indicative of impaired cognitive function [29,30]. These cut-offs were selected for clinical applicability and comparability with previous studies, but should not be considered substitutes for comprehensive neuropsychological evaluation.

### 2.2. Bias and Sample Size

To minimize selection bias, all eligible patients who provided written informed consent were consecutively enrolled during the study period. The use of standardized cognitive assessment tools and internationally accepted criteria for CRS and ICANS grading was intended to reduce measurement bias. No formal a priori sample size calculation was performed. This was an exploratory prospective single-center cohort study, and all consecutive eligible patients treated during the study period were included.

### 2.3. Statistical Analysis

Statistical analysis was performed with RStudio (R version 4.5.1). Categorical variables are presented as frequencies and percentages, whereas continuous variables are presented as mean ± standard deviation or median (interquartile range) as appropriate. The distribution of quantitative variables was assessed with the Shapiro–Wilk test and histogram. MoCA and MMSE total scores were analyzed as continuous variables, while cognitive impairment was treated as a categorical variable based on the predefined cut-off values described above (MoCA ≤ 25 and/or MMSE ≤ 23). Related categorical variables (cognitive impairment) were compared at different time points (T1, T2, T3, T4) with the Cochran’s Q test. In contrast, related quantitative non-normally distributed variables (total MoCA and MMSE scores and individual domain scores) were compared with the Friedman test. In cases where the Friedman test was statistically significant, post hoc Wilcoxon signed-rank tests with Bonferroni correction were performed.

Independent categorical and quantitative variables were compared with the chi-squared or Fisher’s exact test and Student’s t or Mann–Whitney U test, respectively. Univariate logistic regression analysis findings were presented with odds ratios [OR, 95% confidence intervals (95% CI)] and *p*-values. Logarithmic transformation, with natural logarithm (Ln), of non-normally distributed variables was performed when considered necessary. Variables with *p* < 0.10 in univariable analysis were entered into the multivariable model. Multicollinearity among variables included in the multivariable models was assessed using the variance inflation factor (VIF). In all models, VIF values were <2, indicating no relevant multicollinearity. The level of statistical significance was set at *p* < 0.05. Analyses were performed using available data at each time point. Patients with missing cognitive assessments were excluded from the corresponding comparisons. No subgroup or sensitivity analyses were conducted due to the limited sample size.

## 3. Results

### 3.1. Baseline Characteristics of Study Participants

We prospectively studied 36 patients who received CAR-T cell therapy at our center, as presented in Supplementary Figure S1. The median age of participants was 52 (40.75–62.75) years, and 22 (61.1%) were male. The primary indication for CAR-T cell therapy was NHL in 28 (77.8%) cases, while 5 (13.9%) were treated for B-ALL, and 3 (8.3%) for MM. Moreover, the majority of patients underwent CAR-T cell therapy on refractory or active disease [24 (66.7%)]. The median lines of previous treatments were 3 (2–4), whereas 3 (8.3%) had undergone a previous autologous HCT, and 3 (8.3%) an allogeneic HCT. Twenty-three (63.9%) received Axi-cel, six (16.6%) Brexu-cel, four (11.1%) Tisa-cel, and three (8.3%) CAR-T cell products for MM. The clinical characteristics of our population are summarized in Table 1. Furthermore, the laboratory findings of the patients before the administration of lymphodepleting chemotherapy (T1), on the day of CAR-T cell therapy, and during the post-infusion period are presented in Supplementary Table S1.

### 3.2. Toxicities and Survival Outcomes in the Post-Infusion Period

Thirty-three (91.7%) patients developed CRS of any grade, and twenty-three (63.9%) developed ICANS of any grade (Table 2). Specifically, 27 (75%) were classified as CRS grade II–IV, and 13 (36.1%) as ICANS grade II–IV. Concerning the management of toxicities, 33 (91.7%) were treated with tocilizumab, and 23 (63.9%) with corticosteroids. Additionally, during the follow-up, 28 (77.8%) patients were alive, and 8 (22.2%) died. Most deaths were attributed to disease progression or relapse (n = 6), while two deaths were infection-related. Death occurred at a mean of 193.3 (±159.1) days after CAR-T cell infusion (range: 28–477 days).

### 3.3. Cognitive Outcomes

#### 3.3.1. T1: Baseline Pre-Infusion Assessment

**Figure 1:**
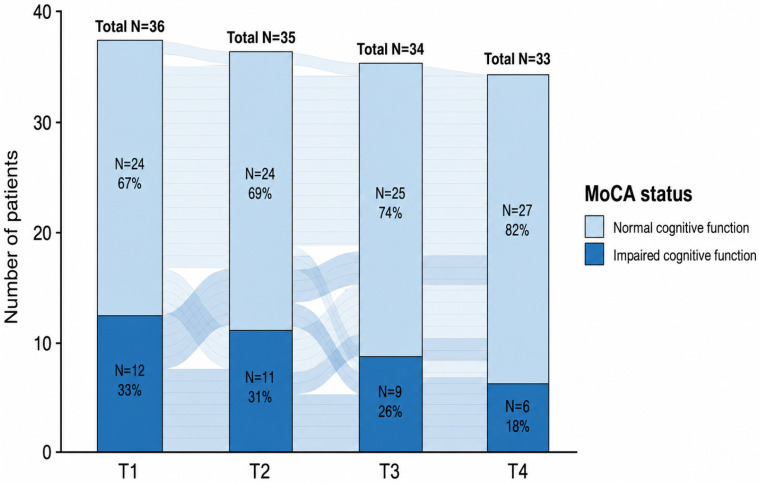
Longitudinal changes in MoCA-defined cognitive status across study time points (Cochran’s Q test *p*-value: 0.112). Shaded flow lines illustrate individual patient transitions between cognitive categories over time. MoCA: Montreal Cognitive Assessment.

**Figure 2:**
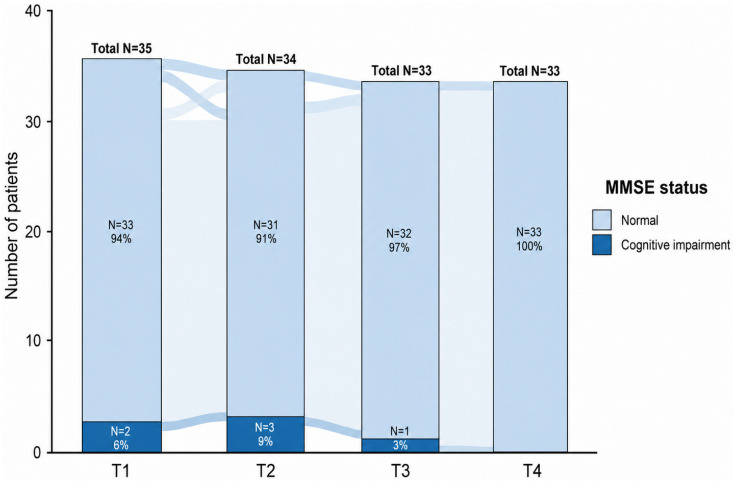
Longitudinal changes in MMSE-defined cognitive status across study time points (Cochran’s Q test *p*-value: 0.172). Shaded flow lines depict individual patient transitions between cognitive status categories over time. MMSE: Mini-Mental State Examination.

Initially, we used the MoCA and MMSE tests to evaluate the cognitive function of our patients before the administration of lymphodepleting chemotherapy to identify pre-infusion cognitive deficits (T1). MoCA measurements were available for 36 patients, and MMSE for 35. The median total MoCA score was 26.5 (25–27.75), while the MMSE total score was 29 (28–29) (Table 3 and Table 4). Moreover, as shown in Table 3 and Table 4, we calculated the median score achieved for each question in both tests. Based on the aforementioned cut-offs, 12/36 (33.3%) had cognitive impairment according to the MoCA test, and 2/35 (5.7%) as defined by MMSE (Figure 1 and Figure 2). Importantly, all patients classified in the cognitive impaired group according to the MMSE tests were also in the same category based on the MoCA test at all time points.

#### 3.3.2. T2: Following CAR-T Cell Infusion

Then, patients were assessed 6 h post CAR-T cell infusion (after the administration of the CAR-T cell product). Thirty-five and thirty-four patients had MoCA and MMSE values available, respectively. The median MoCA total score was 26 (24–27), whereas 11/35 (31.43%) were classified in the category of cognitive impairment (Table 4, Figure 1). For MMSE, its median value was 28 (26–29), with 3/34 (8.88%) having impaired cognition (Table 3, Figure 2).

#### 3.3.3. T3: 3 Months Post-Infusion

Subsequently, the patients were evaluated 3 months after infusion (Τ3). Based on the MoCA test, 9/34 (26.47%) were categorized as cognitively impaired, while 1/33 (3%) were on the MMSE test (Figure 1 and Figure 2). Specifically, the median MoCA score was 26 (24–28), and MMSE was 29 (27–29) (Table 3 and Table 4). Among these patients, 7/9 who had cognitive impairment according to MoCA values also had cognitive defects at T1, and 5/9 at T2. The detailed characteristics are presented in Supplementary Table S2.

#### 3.3.4. T4: 6 Months Post-Infusion

Finally, study participants were assessed at 6 months post-infusion. Particularly, 6/33 (18.18%) had cognitive impairment on the MoCA test [median: 26 (25–27)], and none out of 33 in MMSE [median: 28 (28–29)] (Table 3 and Table 4, Figure 1 and Figure 2). All of these 6 patients had cognitive impairment at T1 and T3, and 5/6 at T2 (Supplementary Table S3).

### 3.4. Comparison of Patients’ Cognitive Outcomes at Different Time Points

**Figure 3:**
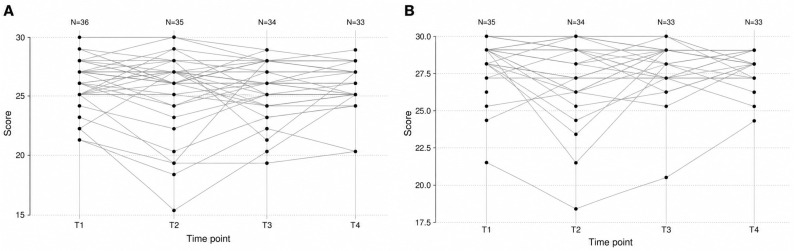
Spaghetti plots for individual longitudinal trajectories of MoCA (*p* = 0.672) (**A**) and MMSE (*p* = 0.130) (**B**) total scores across study time points. The *Y*-axis represents the corresponding total cognitive test score, with higher scores indicating better performance. MMSE: Mini-Mental State Examination; MoCA: Montreal Cognitive Assessment.

To further assess within-patient changes of cognitive function at different time points (T1, T2, T3, T4), we used the Cochran’s Q test comparisons. Nevertheless, Cochran’s Q test did not demonstrate a statistically significant overall difference across the four time points for both MoCA (*p* = 0.112) and MMSE (*p* = 0.172) cognitive classification (Figure 1 and Figure 2). Additionally, we compared the within-patient change over time of MoCA and MMSE total scores and domain scores, with the Friedman test for all time points, as shown in Table 3 and Table 4 and Figure 3. Regarding the MoCA domains, we found that the abstraction domain was statistically significant across the various time periods (*p* = 0.046) (Table 4 and Supplementary Figure S2). However, none of the pairwise comparisons in the post hoc paired Wilcoxon signed-rank analysis remained significant after Bonferroni correction. Regarding MMSE domains, attention and calculation were statistically significantly different among all time points (*p* = 0.032) and, specifically, decreased from T1 to T2 (*p* = 0.033) (Table 3 and Supplementary Figure S3). Similarly, we report that the language domain changed over time (*p* = 0.041), but the pairwise comparisons in the post hoc analysis were not significant (Table 3 and Supplementary Figure S4).

### 3.5. Factors Associated with Cognitive Dysfunction

#### 3.5.1. Cognitive Impairment at T1

Initially, we compared the clinical and laboratory characteristics of patients who had cognitive dysfunction (as determined by the MoCA test at T1) before infusion with those who did not (Supplementary Table S4). We noted that the mean age of patients with cognitive impairment was higher [56.83 (±9.907) versus (vs) 46.5 (±17.547), *p* = 0.031]. Furthermore, they had lower CRP values [0.41 (0.17–0.75) vs. 0.89 (0.65–3.64), *p* = 0.016]. The remaining comparisons were not statistically significant and are presented in Supplementary Table S4. In addition, the prevalence of cognitive impairment, median MoCA, and MMSE at T1 were comparable among male and female patients, NHL and non-NHL patients, those with a previous history of HCT and those without, and Axi-cel recipients vs. the rest (Supplementary Table S5). In univariate logistic regression analysis, cognitive impairment at T1 was associated with CRP (LnCRP) at the same time point (OR: 0.476, 95% CI: 0.215–0.861, *p* = 0.0315) (Supplementary Table S6). After adjusting for age, the association with CRP did not remain significant (*p* = 0.076) (Supplementary Table S7).

#### 3.5.2. Cognitive Impairment at T2

We then conducted comparisons between CAR-T cell recipients who had cognitive impairment at T2 and the rest of the study population (Supplementary Table S8). The mean age of patients with cognitive dysfunction was higher [60.64 (±6.9) vs. 45.21 (±17.1), *p* < 0.001], along with decreased CRP [0.25 (0.13–0.8) vs. 0.92 (0.58–3.64), *p* = 0.004] values obtained at baseline. Additionally, they had elevated platelet levels on the day of infusion [185.09 (±69.5) vs. 127.71 (±60.6), *p* = 0.018]. Importantly, as expected, the prevalence of cognitive dysfunction [7/11 (63.6%) vs. 5/24 (20.8%), *p* = 0.022] was higher at baseline (T1), while the median MoCA [25 (22–26) and 27 (26–28), *p* < 0.001] and MMSE [28 (28–29) vs. 29 (29–30), *p* = 0.021] total scores were lower in patients with cognitive impairment at T2 compared to those without. Moreover, no differences were observed in the prevalence of cognitive impairment at T2 according to sex, disease (NHL vs. non-NHL), prior HCT history, or axi-cel treatment compared with other CAR-T products (Supplementary Table S9).

In univariate analysis, cognitive impairment at T2 was associated with age (OR: 1.1, 95% CI: 1.03–1.2, *p* = 0.0211), CRP (LnCRP) levels at baseline (OR: 0.342, 95%CI: 0.123–0.702, *p* = 0.0136), platelets at day 0 (OR: 1.02, 95% CI: 1–1.03, *p* = 0.0326), cognitive impairment at T1 (OR: 6.65, 95% CI: 1.45–35.6, *p* = 0.0184), and MoCA total score at baseline (OR: 0.479, 95% CI: 0.249–0.746, *p* = 0.00624) (Supplementary Table S10). However, in multivariate analysis models adjusted for age and cognitive impairment at T1, CRP at baseline was not associated with cognitive impairment at T2 (*p* = 0.072), whereas platelets at day 0 were still associated (OR: 1.02, 95% CI: 1.003–1.043, *p* = 0.047), but not independently from age (Supplementary Table S11).

#### 3.5.3. Cognitive Impairment at T3 and T4

We report that patients with cognitive impairment at T3 had lower median max CRP levels post-infusion in comparison to those without [2.39 (1.72–3.9) vs. 6.13 (2.35–12.21), *p* = 0.027] (Supplementary Table S12). Moreover, those patients in the baseline had a higher prevalence of impaired cognitive function [7/9 (77.8%) vs. 5/25 (20%), *p* = 0.04] and lower MoCA total score [23 (21.5–25.5) vs. 27 (26–28), *p* = 0.02]. Similarly, they were characterized by lower MMSE at T2 [26 (22–27.5) vs. 29 (27–30), *p* = 0.002]. The analysis based on gender, CAR-T cell indication (NHL vs. other), previous history of HCT, and CAR-T cell production (axi-cel vs. other) did not identify any statistically significant findings (Supplementary Table S13). We did not perform univariate analysis for cognitive impairment at T3. Furthermore, due to the small number of patients with cognitive dysfunction at T4 (n = 6), we did not further analyze their characteristics.

### 3.6. Comparison of Cognitive Outcomes Between Patients With and Without Toxicities

Subsequently, we compared the prevalence of cognitive impairment, median MoCA, and total MMSE score at T1, T2, T3, and T4 between ICANS and non-ICANS patients, as well as among those with ICANS ≥ II and those without. However, we did not identify any statistically significant differences (Supplementary Tables S14 and S15). Similarly, the cognitive outcomes were not different among patients with CRS ≥ II and those with CRS grade I or no CRS (Supplementary Table S16).

Finally, we report the cognitive outcomes of patients with grade III–IV toxicities. Specifically, we found that the cognitive function at baseline and at T2, T3, and T4 was comparable among CAR-T cell recipients with ICANS grade III–IV (n = 6) and the rest of the study population (Supplementary Table S17). As mentioned above, CRS grade III–IV was observed in three patients, who all had normal cognitive function at baseline, while data after infusion were available for two of them, and both of them presented normal cognitive function at T2, T3, and T4 (Supplementary Table S18).

## 4. Discussion

Previous studies have evaluated cognitive outcomes after CAR-T cell therapy using patient-reported measures, neuropsychological testing, or shorter-term assessments. Building on this literature, our study provides prospective real-world longitudinal data using two simple bedside cognitive screening tools, MoCA and MMSE, applied at predefined time points before lymphodepletion, early after infusion, and at 3 and 6 months post-infusion. In this cohort, 36 patients who underwent CAR-T cell therapy (63.9% Axi-cel recipients) for various underlying diagnoses, primarily R/R NHL, were included. Regarding the prevalence and severity of infusion-related toxicities, our findings are consistent with those of other international studies [36,37].

Specifically, 33/36 (91.7%) developed CRS of any grade, whereas 23 (63.9%) developed ICANS. Grade III–IV CRS was reported in 3/36 (8.3%), and grade III–IV ICANS in 6/36 (16.7%). In various clinical trials, the all-grade CRS incidence ranges from 30 to 90%, and all-grade ICANS affects approximately 40–65% of patients receiving CAR-T cells, which is consistent with our findings [4,38]. However, the ICANS rate in this cohort is higher than that reported by Mohn et al., who observed 4/15 ICANS-affected patients during a 10-day follow-up period post CAR-T administration [27]. This difference may be explained by the shorter follow-up period. Additionally, in a previously published multicenter Greek study by our group, severe ICANS (grade ≥ III) was reported in 14/175 (8%) CAR-T cell patients and severe CRS (grade ≥ III) in 17/175 (9.7%) individuals [13]. Moreover, during the 6-month follow-up, 28 (77.8%) patients were still alive, and 8 (22.2%) died. In the recent systematic review and meta-analysis of CAR-T cell patients regarding non-relapse mortality, Cordas dos Santos et al. reported 7.5% of non-relapse deaths, with approximately half of them attributed to infections, followed by other malignancies (7.8%) and cardiovascular/respiratory complications (7.3%) [39].

Concerning baseline cognitive impairment (T1) before the administration of lymphodepleting chemotherapy, 12/36 (33.33%) of the patients experienced impaired baseline cognitive function, based on the MoCA test. Furthermore, cognitive impairment at T1 was associated with older age and lower CRP levels at the same time point. Nevertheless, the association with CRP did not remain significant after the adjustment for age. Age-related changes in cognitive reserve and baseline neurological vulnerability may partially explain the increased prevalence of cognitive deficits observed in older individuals. Additionally, the association with lower inflammatory markers may reflect complex interactions between systemic inflammation, disease burden, and baseline patient characteristics. However, the biological mechanisms underlying this finding remain unclear. Future prospective studies with larger cohorts and detailed neurocognitive and biomarker assessments are crucial to validate these observations and further elucidate the mechanisms linking baseline inflammatory status with cognitive function in CAR-T cell recipients. Similar rates of baseline cognitive impairment have also been reported in other prospective studies. For instance, Kuznetsova et al. assessed cognitive function before CAR-T cell infusion in 60 patients using multiple neurocognitive tools, including the MoCA test, and reported baseline cognitive impairment in 27% of participants [40]. However, cognitively impaired patients in that cohort were more frequently younger and diagnosed with B-ALL, highlighting potential differences in patient populations, disease characteristics, and treatment history that may influence baseline cognitive performance across studies.

Following infusion (T2), 11/35 (31.4%) of the participants had cognitive dysfunction based on the MoCA test. Older age, lower CRP at baseline, and the presence of baseline impaired cognitive function (at T1) were associated with cognitive impairment at T2 in univariate analysis. However, in multivariate analysis, CRP at baseline was not associated with cognitive impairment (in a model adjusted for age and cognitive impairment at T1). In the study by Nie et al., which focused on brexu-cel in patients with MCL, severe ICANS was associated with more pronounced anemia and thrombocytopenia, higher peak CRP levels, and acute brain magnetic resonance imaging (MRI) abnormalities during the clinical neurotoxicity period, including T2/FLAIR (fluid-attenuated inversion recovery) hyperintensities, non-vascular diffusion-weighted imaging (DWI) abnormalities, asymmetric hyperemia on arterial spin labeling sequences, worsening of pre-existing white matter changes, and optic nerve DWI signal changes [41]. The association of these markers with cognitive outcomes should be investigated in future studies.

At three months post-CAR-T cell infusion (T3), 9/34 (26.5%) exhibited cognitive impairment. Specifically, the majority of them (7/9) also experienced baseline (at T1) cognitive deficits. Additionally, we found that 6/33 (18.2%) patients still presented with cognitive dysfunction at six months following infusion (T4). Therefore, this finding may be explained by the participants’ established mild cognitive impairment, as evidenced by their MoCA and MMSE scores pre-infusion (T1). These results are consistent with previous studies by Levine et al. [42] and Shalabi et al. [43]. Notably, the frequency of MoCA-defined cognitive impairment was numerically lower at 3 and 6 months than at the early post-infusion assessment, suggesting that early cognitive changes may be at least partly transient and potentially reversible in a substantial proportion of patients. Importantly, all patients with cognitive impairment at 6 months had already shown impairment at baseline, indicating that persistent deficits may largely reflect pre-existing vulnerability rather than a sustained de novo post-infusion effect.

In our cohort, the prevalence of cognitive dysfunction and total MoCA and MMSE scores at all the time points under study was not different between patients with ICANS and those without ICANS, ICANS grade ≥ II, the rest of the study population, and ICANS grade III–IV and those without ICANS or ICANS I–II. Similarly, Ursu and colleagues reported that no significant association was observed between baseline cognitive performance and the onset of ICANS (*p* = 0.57) [44]. Additionally, MoCA scores did not vary according to ICANS grade. In contrast to our findings, Hernández-Tost et al. found that a MoCA score < 26 at the time of CAR T-cell infusion was significantly associated with a higher risk of grade III–IV neurotoxicity (*p* = 0.04, OR: 12) in their exploratory analysis [45]. However, in this study, only patients with isolated central nervous system relapse of B-cell lymphomas were included.

This study highlights the importance of cognitive assessment before CAR-T cell therapy, as patients with pre-existing cognitive impairment may be at increased risk of developing cognitive dysfunction following infusion compared with individuals without baseline deficits. Total MoCA and MMSE scores and the prevalence of cognitive impairment did not differ significantly among the aforementioned time intervals. Interestingly, we observed that the abstraction domain showed variation across the different time points in the MoCA assessment. Moreover, within the MMSE domains, attention and calculation differed significantly over time, with a decrease observed from T1 to T2, while the language domain also demonstrated fluctuations during follow-up. These findings may indicate transient alterations in higher cognitive functions, particularly attention and executive processes, which may be vulnerable to the inflammatory and neurotoxic effects associated with CAR-T cell therapy. Nevertheless, the clinical relevance of these domain-specific changes remains unclear and should be further explored in larger prospective studies.

This study has several limitations. First, the relatively small sample size may have limited the statistical power to detect subtle longitudinal changes or subgroup-specific associations. Second, the study population included patients treated with different CAR-T cell products for heterogeneous hematologic malignancies, which may introduce clinical variability. Third, repeated administration of cognitive tests may have been influenced by a potential practice effect, which could partially affect longitudinal cognitive performance. In addition, the absence of a non-CAR-T comparator group limits the ability to distinguish treatment-related cognitive changes from those related to the underlying disease or prior therapies. Moreover, the use of predefined MoCA and MMSE cut-offs may have influenced the classification of cognitive impairment, as these thresholds may not fully account for differences in age, education, language, cultural background, or race/ethnicity. Although we applied the official MoCA education correction, residual misclassification remains possible. Therefore, the reported rates of cognitive impairment should be interpreted as screening-based estimates rather than definitive neuropsychological diagnoses. Finally, an additional limitation is the lack of prospectively collected structured neurological examinations and quality-of-life assessments, which could provide a more comprehensive evaluation of survivorship outcomes after CAR-T cell therapy.

Future research should focus on validating these findings in larger, multicenter cohorts with longer follow-up periods. Integrating neurocognitive assessments with biological markers of inflammation and endothelial activation, as well as advanced monitoring approaches such as EEG and neuroimaging techniques, including MRI, may provide further insights into the mechanisms underlying cognitive changes after CAR-T cell therapy. Additionally, incorporating patient-reported outcomes and quality-of-life measures will be important to better understand the real-world impact of neurocognitive alterations in CAR-T cell recipients.

## 5. Conclusions

In this prospective cohort of adult CAR-T cell recipients, overall cognitive performance remained largely stable during the 6-month follow-up period. However, baseline cognitive impairment was common and was associated with older age and early post-infusion cognitive dysfunction. MoCA appeared more sensitive than MMSE in detecting mild cognitive deficits in this population. These findings support the incorporation of simple longitudinal cognitive assessments into routine clinical monitoring of CAR-T recipients and highlight the potential value of pre-infusion cognitive screening. Further multicenter studies are crucial to better define the mechanisms, predictors, and long-term clinical significance of neurocognitive changes following CAR-T cell therapy.

## Figures and Tables

**Table 1:** Baseline clinical characteristics of the study participants (N = 36).

**Median age (IQR)**	52 (40.75–62.75)
**Gender, n (%)**	
Male	22 (61.1)
Female	14 (38.9)
**Disease, n (%)**	
NHL	28 (77.8)
B-ALL	5 (13.9)
MM	3 (8.3)
**Disease phase, n (%)**	
CR	5 (13.9)
Refractory/active	24 (66.7)
Relapsed	7 (19.4)
**Median number of previous treatment lines (IQR) ^1^**	3.00 (2.00–4.00)
**Previous HCT, n (%)**	
No	30 (83.4)
Auto	3 (8.3)
Allo	3 (8.3)
**CAR-T cell product, n (%)**	
Axicabtagene ciloleucel	23 (63.9)
Brexucabtagene autoleucel	6 (16.6)
Tisagenlecleucel	4 (11.1)
Ciltacabtagene autoleucel	1 (2.8)
PHE-885	2 (5.6)

^1^ Two patients had missing data. Allo: allogeneic; Auto: autologous; B-ALL: B-cell acute lymphoblastic leukemia; CAR-T: chimeric antigen receptor T-cell; CR: complete remission; HCT: hematopoietic cell transplantation; IQR: interquartile range; MM: multiple myeloma; NHL: non-Hodgkin lymphoma.

**Table 2:** Toxicity and management characteristics of CAR-T cell recipients.

**CRS, n (%)**	
Grade I	6 (16.7)
Grade II	24 (66.6)
Grade III	1 (2.8)
Grade IV	2 (5.6)
**Grade ≥ II CRS, n (%)**	27 (75.0)
**ICANS, n (%)**	
Grade I	10 (27.8)
Grade II	7 (19.4)
Grade III	4 (11.1)
Grade IV	2 (5.6)
**Grade ≥ II ICANS, n (%)**	13 (36.1)
Tocilizumab ^1^, n (%)	33 (91.7)
Corticosteroids, n (%)	23 (63.9)

^1^ Tocilizumab was administered at a dose of 8 mg/kg according to institutional practice and standard toxicity management protocols. CAR-T: chimeric antigen receptor T-cell; CRS: cytokine release syndrome; ICANS: immune effector cell-associated neurotoxicity syndrome.

**Table 3:** Longitudinal changes in MMSE domains and total score across study time points. We performed the post hoc paired Wilcoxon signed-rank with Bonferroni correction in comparisons found statistically significant in the Friedman test.

Median (IQR25–75)	T1 (N = 35)	T2 (N = 34)	T3 (N = 33)	T4 (N = 33)	*p*-Value ^1^
Orientation	10 (10–10)	10 (10–10)	10 (10–10)	10 (10–10)	0.246
Registration	3 (3–3)	3 (3–3)	3 (3–3)	3 (3–3)	0.194
Attention and calculation	5 (5–5)	5 (4–5)	5 (4.5–5)	5 (4.5–5)	0.032, 0.033 ^2^
Recall	2 (1–2)	2 (1–2)	2 (1.5–2)	2 (1–2)	0.471
Language	9 (9–9)	9 (9–9)	9 (9–9)	9 (9–9)	0.041 ^3^
Total score	29 (28–29)	28 (26–29)	29 (27–29)	28 (28–29)	0.130

^1^ Friedman *p*-value; when applicable, post hoc Bonferroni-corrected Wilcoxon *p*-values are also reported. ^2^ Wilcoxon signed-rank test with Bonferroni correction for T1 versus T2 scores comparisons *p*-value. ^3^ Although the overall Friedman test was significant, none of the pairwise comparisons remained significant after Bonferroni correction. MMSE: Mini-Mental State Examination.

**Table 4:** Longitudinal changes in MoCA domains and total scores across study time points.

Median Score (IQR25–75)	T1 (N = 36)	T2 (N = 35)	T3 (N = 34)	T4 (N = 33)	*p*-Value ^1^
Visuospatial/executive function	4.5 (4–5)	4 (4–5)	5 (4–5)	5 (4–5)	0.057
Naming	3 (3–3)	3 (3–3)	3 (3–3)	3 (3–3)	0.572
Attention	6 (6–6)	6 (5–6)	6 (5–6)	6 (5–6)	0.140
Language	3 (2.25–3)	3 (2–3)	3 (2–3)	3 (2–3)	0.606
Abstraction	2 (2–2)	2 (2–2)	2 (2–2)	2 (2–2)	0.046 ^2^
Delayed recall	2 (1.25–3.75)	3 (2–3)	2 (2–3)	2 (1–3)	0.566
Orientation	6 (6–6)	6 (6–6)	6 (6–6)	6 (6–6)	0.096
Total score	26.5 (25–27.75)	26 (24–27)	26 (24–28)	26 (25–27)	0.672

^1^ Friedman *p*-value. ^2^ Although the overall Friedman test was significant, none of the pairwise comparisons in the post hoc paired Wilcoxon signed-rank analysis remained significant after Bonferroni correction. The other comparisons were not statistically significant. MoCA: Montreal Cognitive Assessment.

## Data Availability

Data will be readily available upon request to the corresponding author.

## Supplementary Materials

The following supporting information can be downloaded at: https://www.mdpi.com/article/10.3390/cancers18111803/s1, Figure S1: Flow diagram of study participants included at each time point and available cognitive test values. MMSE: Mini-Mental State Examination, MoCA: Montreal Cognitive Assessment, T1: baseline assessment time point, T2: post–CAR-T cell infusion assessment time point, T3: 3-month post-infusion assessment time point, T4: 6-month post-infusion assessment time point. Figure S2: Spaghetti plot for individual longitudinal trajectories of MoCA abstraction domain scores across study time points (Friedman test *p* = 0.046). The Y-axis represents the MoCA abstraction score (range: 0–2), with higher scores indicating better performance. All patients with available scores were included, regardless of CAR-T cell product; no product-specific subgroup analysis is shown. MoCA: Montreal Cognitive Assessment; CAR-T: chimeric antigen receptor T-cell. Figure S3: Spaghetti plot for individual longitudinal trajectories of MMSE attention and calculation domain scores across study time points (Friedman test *p* = 0.032). The Y-axis represents the MMSE attention/calculation score (range: 0–5), with higher scores indicating better performance. A post-hoc Wilcoxon signed-rank test with Bonferroni correction showed a decrease from T1 to T2 (*p* = 0.033). All patients with available scores were included, regardless of CAR-T cell product; no product-specific subgroup analysis is shown. MMSE: Mini-Mental State Examination; CAR-T: chimeric antigen receptor T-cell. Figure S4: Spaghetti plot for individual longitudinal trajectories of MMSE language scores across study timepoints (*p* = 0.041). MMSE: Mini-Mental State Examination. Table S1: Laboratory characteristics of patients and endothelial injury indices of study participants at baseline (T1, before the administration of lymphodepleting chemotherapy), day 0 (day of CAR-T cell infusion), and post-infusion. Table S2: Detailed characteristics of patients with neurocognitive dysfunction at T3 (N = 9). Table S3: Detailed characteristics of patients with neurocognitive dysfunction at T4 (N = 6).Table S4: Baseline characteristics and inflammatory markers based on cognitive status at baseline (T1). Table S5: Cognitive outcomes at baseline (T1), stratified by gender, underlying diagnosis, prior HCT, and CAR-T product type. Table S6: Univariate logistic regression analysis of factors associated with cognitive impairment, assessed by the MoCA test at T1 following CAR-T cell infusion. Table S7: Multivariate logistic regression analysis models for the presence of cognitive impairment at T1 (baseline), assessed by the MoCA test. Variables with *p*-value < 0.1 in univariate logistic regression analysis (Supplementary Table S6) were included. Moreover, the variables included were checked for multicollinearity by calculating VIF. In all cases, VIF was less than 2. Table S8: Baseline and infusion characteristics, inflammatory markers, and cognitive outcomes according to cognitive status after CAR-T cell infusion (T2). Table S9: Cognitive outcomes after CAR-T cell infusion (T2), stratified by gender, underlying diagnosis, prior HCT, and CAR-T product type. Table S10: Univariate logistic regression analysis of factors associated with cognitive impairment, assessed by the MoCA test at T2 following CAR-T cell infusion. Table S11: Multivariate logistic regression analysis models for the presence of cognitive impairment at T2, assessed by the MoCA test. Variables with *p*-value < 0.1 in univariate logistic regression analysis (Supplementary Table S10) were included. Moreover, the variables included were checked for multicollinearity by calculating VIF. In all cases, VIF was less than 2. Table S12: Baseline and infusion characteristics, inflammatory markers, treatment-related toxicities, and cognitive outcomes according to cognitive status at 3 months following CAR-T cell infusion (T3). Table S13: Cognitive outcomes at 3 months following CAR-T cell infusion (T3), stratified by gender, underlying diagnosis, prior HCT, and CAR-T product type. Table S14: Comparison of cognitive impairment prevalence, median MoCA, and median MMSE total score between patients with ICANS and those without at T1, T2, T3, and T4. Table S15: Comparison of cognitive impairment prevalence, median MoCA, and median MMSE total score between patients with ICANS ≥ II and the rest of the study participants at T1, T2, T3, and T4. Table S16: Comparison of cognitive impairment prevalence, median MoCA, and median MMSE total score between patients with CRS ≥ II and the rest of the study participants at T1, T2, T3, and T4. Table S17: Comparison of cognitive impairment prevalence, median MoCA, and median MMSE total score between patients with ICANS grade III–IV and the rest of the study participants at T1, T2, T3, and T4. Table S18: Cognitive outcomes in patients with CRS grade III–IV.

## Abbreviations

The following abbreviations are used in this manuscript:

Axi-cel	Axicabtagene ciloleucel
B-ALL	B-cell acute lymphoblastic leukemia
BCMA	B-cell maturation antigen
Brexu-cel	Brexucabtagene autoleucel
CAR-T	Chimeric antigen receptor T
CI	Confidence interval
CRP	C-reactive protein
CRS	Cytokine release syndrome
DLBCL	Diffuse large B-cell lymphoma
EASIX	Endothelial Activation and Stress Index
EBMT	European Society for Blood and Marrow Transplantation
EEG	Electroencephalography
HCT	Hematopoietic cell transplantation
ICANS	Immune effector cell-associated neurotoxicity syndrome
ICE	Immune effector cell-associated Encephalopathy score
IL	Interleukin
MCL	Mantle cell lymphoma
MM	Multiple myeloma
MMSE	Mini-Mental State Examination
MoCA	Montreal Cognitive Assessment
MRI	Magnetic resonance imaging
Liso-cel	Lisocabtagene maraleucel
NHL	Non-Hodgkin lymphoma
OR	Odds ratio
R/R	Relapsed/refractory
Tisa-cel	Tisagenlecleucel
