# Supplementary material for: Longitudinal Cognitive Assessment After CAR-T Cell Immunotherapy: A Prospective Cohort Study
Source: Cancers (Basel). 2026 Jun 1;18(11):1803. doi: 10.3390/cancers18111803 (PMC13255979; doi:10.3390/cancers18111803)
Supplement: Supplementary file 1 [file cancers-18-01803-s001.zip › Supplementary figures.pdf]

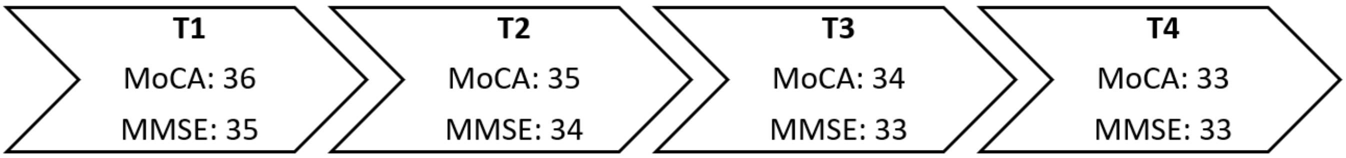

Supplementary Figure S1. Flow diagram of study participants included at each time point and available cognitive test values. MMSE: Mini-Mental State Examination, MoCA: Montreal Cognitive Assessment, T1: baseline assessment time point, T2: post–CAR-T cell infusion assessment time point, T3: 3-month post-infusion assessment time point, T4: 6-month post-infusion assessment time point.

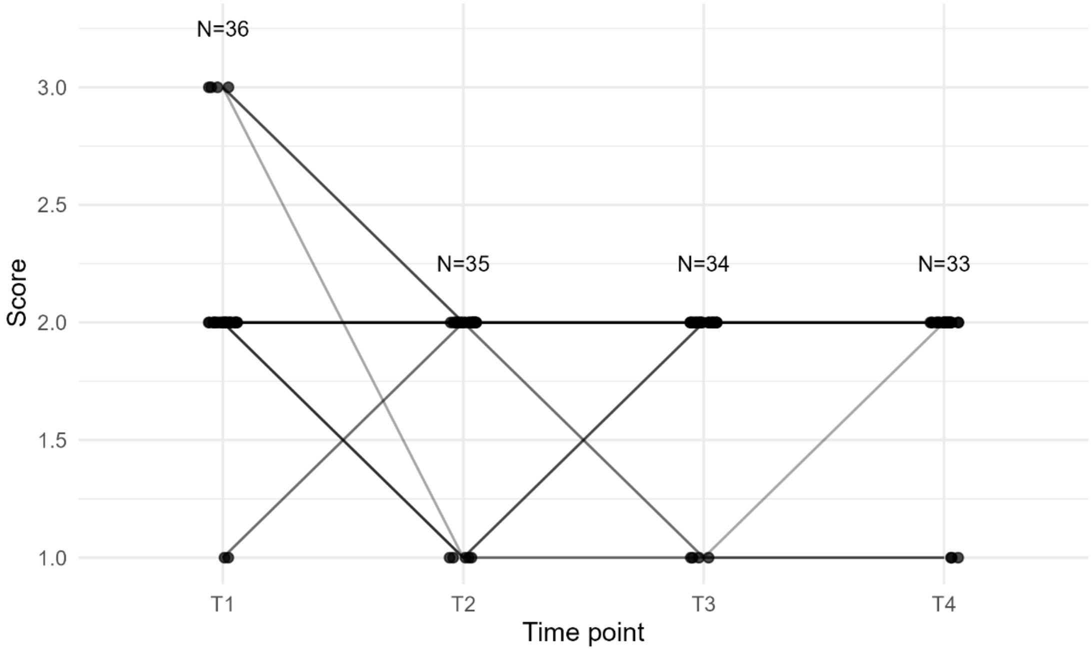

Supplementary Figure S2. Spaghetti plot for individual longitudinal trajectories of MoCA abstraction domain scores across study time points (Friedman test $p = 0.046$). The Y-axis represents the MoCA abstraction score (range: 0–2), with higher scores indicating better performance. All patients with available scores were included, regardless of CAR-T cell product; no product-specific subgroup analysis is shown. MoCA: Montreal Cognitive Assessment; CAR-T: chimeric antigen receptor T-cell.

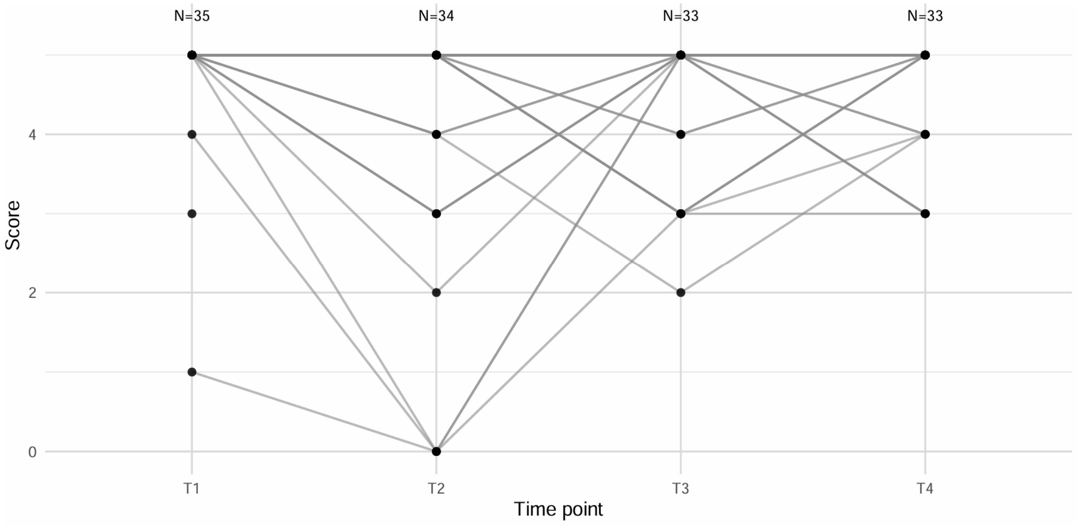

Supplementary Figure S3. Spaghetti plot for individual longitudinal trajectories of MMSE attention and calculation domain scores across study time points (Friedman test $p = 0.032$). The Y-axis represents the MMSE attention/calculation score (range: 0–5), with higher scores indicating better performance. A post-hoc Wilcoxon signed-rank test with Bonferroni correction showed a decrease from T1 to T2 ($p = 0.033$). All patients with available scores were included, regardless of CAR-T cell product; no product-specific subgroup analysis is shown. MMSE: Mini-Mental State Examination; CAR-T: chimeric antigen receptor T-cell.

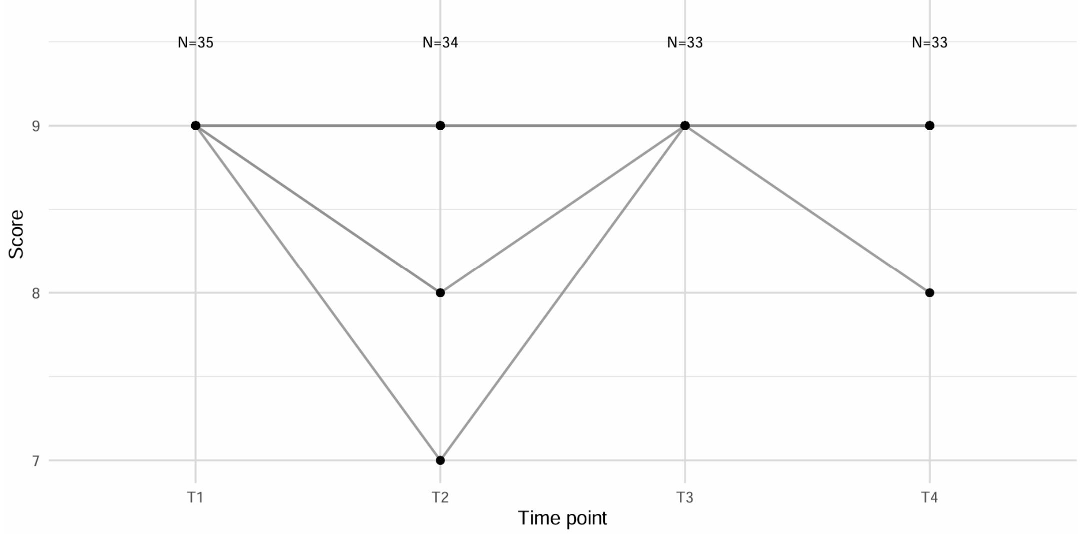

Supplementary Figure S4. Spaghetti plot for individual longitudinal trajectories of MMSE language scores across study timepoints ($p = 0.041$). MMSE: Mini-Mental State Examination.
