# Supplementary material for: Longitudinal Cognitive Assessment After CAR-T Cell Immunotherapy: A Prospective Cohort Study
Source: Cancers (Basel). 2026 Jun 1;18(11):1803. doi: 10.3390/cancers18111803 (PMC13255979; doi:10.3390/cancers18111803)
Supplement: Supplementary file 1 [file cancers-18-01803-s001.zip › Supplementary Tables.pdf]

Supplementary Table S1. Laboratory characteristics of patients and endothelial injury indices of study participants at baseline (T1, before the administration of lymphodepleting chemotherapy), day 0 (day of CAR-T cell infusion), and post-infusion.

Median ferritin at baseline (IQR)	298 (72-584.75)
Mean platelets baseline (±SD)	170.50 (±89.71)
Median creatinine baseline (IQR)	0.71(0.56-0.94)
Median LDH baseline (IQR)	227 (187.25-466.25)
Median IL-6 baseline (IQR)[2]	24.5 (10-43.5)
Median CRP baseline (IQR)	0.75 (0.38-1.75)
Mean platelets day 0 (±SD)	142.94 (±69.18)
Median creatinine day 0 (IQR)	0.68 (0.54-0.87)
Median LDH day 0 (IQR)	226 (180.25-390.5)
Median peak CAR-T cells post-infusion (IQR)[1]	13.25 (1.835-60.25)
Median platelets day +14 (IQR)	143.50 (32.25-229.5)
Median creatinine day +14 (IQR)	0.68 (0.54-0.99)
Median LDH day +14 (IQR)	229.5 (166.5-320.5)
Median max IL-6 post-infusion (IQR)	1197 (142-3059)
Median IL-6 three days post IL-6 max values (IQR)[3]	76 (29-167)
Median IL-6 day +14 (IQR)[4]	44 (24-78)
Median CRP max (IQR)	4.29 (2.3-10.47)

Median CRP three days post CRP max values (IQR)	0.96 (0.44-1.95)
Median CRP day +14 (IQR)	0.15 (0.04-0.34)

[1]Six patients had missing data, [2]Two patients had missing data, [3]Onepatient had missing data, [4]One patient had missing data, CAR-T: chimeric antigen receptor T-cell, CRP: C-reactive protein, IL-6: interleukin-6, IQR: interquartile range, LDH: lactate dehydrogenase, SD: standard deviation

Supplementary Table S2. Detailed characteristics of patients with neurocognitive dysfunction at T3 (N = 9).

Patient No	Age at infusion	CAR-T cell indication	CAR-T cell product	MoCA cognitive status at baseline	MoCA cognitive status after infusion	MoCA cognitive status 6 months post-infusion
1	22	NHL	Axi-cel	Normal	Normal	Normal
2	64	NHL	Axi-cel	Impaired	Impaired	Impaired
3	54	NHL	Axi-cel	Impaired	Impaired	Impaired
4	52	NHL	Axi-cel	Impaired	Impaired	Impaired
5	57	NHL	Brexu-cel	Impaired	Impaired	Impaired
6	58	NHL	Brexu-cel	Impaired	Normal	Normal
7	62	NHL	Axi-cel	Impaired	Normal	Impaired
8	63	NHL	Axi-cel	Impaired	Impaired	Impaired
9	44	ALL	Brexu-cel	Normal	Normal	Normal

ALL: acute lymphoblastic leukemia, Axi-cel: axicabtagene ciloleucel, Brexu-cel: brexucabtagene autoleucel, CAR-T: chimeric antigen receptor T-cell, MoCA: Montreal Cognitive Assessment, NHL: non-Hodgkin lymphoma.

Supplementary Table S3. Detailed characteristics of patients with neurocognitive dysfunction at T4 (N = 6).

Patient No	Age at infusion	CAR-T cell indication	CAR-T cell product	MoCA cognitive status at baseline	MoCA cognitive status after infusion	MoCA cognitive status at 3 months post-infusion
1	64	NHL	Axi-cel	Impaired	Impaired	Impaired
2	54	NHL	Axi-cel	Impaired	Impaired	Impaired
3	52	NHL	Axi-cel	Impaired	Impaired	Impaired
4	57	NHL	Brexu-cel	Impaired	Impaired	Impaired
5	62	NHL	Axi-cel	Impaired	Normal	Impaired
6	63	NHL	Axi-cel	Impaired	Impaired	Impaired

Axi-cel: axicabtagene ciloleucel, Brexu-cel: brexucabtagene autoleucel, CAR-T: chimeric antigen receptor T-cell, MoCA: Montreal Cognitive Assessment, NHL: non-Hodgkin lymphoma.

Supplementary Table S4. Baseline characteristics and inflammatory markers based on cognitive status at baseline (T1).

	Normal cognitive function at baseline (N=24)	Cognitive impairment at baseline (N=12)[1]	p-value
Mean age (±SD)	46.5 (±17.547)	56.83 (±9.907)	0.031
Gender, n (%)<br>Male<br>Female	 16 (66.7)<br>8 (33.3)	 6 (50)<br>6 (50)	0.471
Disease, n (%)<br>NHL<br>ALL<br>MM	 18 (75)<br>4 (16.7)<br>2 (8.3)	 10 (83.3)<br>1 (8.3)<br>1 (8.3)	0.8345
Disease phase, n (%)<br>CR<br>Refractory/ active<br>Relapsed	 4 (16.7)<br>15 (62.5)<br>5 (20.8)	 1(8.3)<br>9 (75)<br>2 (16.7)	0.8766

Median lines of previous treatment (IQR)	3 (2-4)[2]	3 (2-4)[2]	0.800
Previous HCT, n (%)<br>No<br>Auto<br>Allo	 20 (83.3)<br>2 (8.3)<br>2 (8.3)	 10 (83.3)<br>1 (8.3)<br>1 (8.3)	1
CAR-T cell product, (%)<br>Axicabtagene ciloleucel<br>Brexucabtagene autoleucel<br>Tisagenlecleucel<br>Ciltacabtagene autoleucel<br>PHE-885	 15 (62.5)<br>3 (12.5)<br>4 (16.7)<br>1 (4.2)<br>1 (4.2)	 8 (66.7)<br>3 (25)<br>0<br>0<br>1 (8.3)	0.529
Median ferritin (IQR)	347.5 (189.0-584.75)	176.5 (62.25-992.875)	0.237
Median CRP (IQR)	0.89 (0.65-3.64)	0.41 (0.17-0.75)	0.016
Mean Platelets (±SD)	165.75 (±97.149)	180 (±75.660)	0.728
Median creatinine (IQR)	0.64 (0.50-0.99)	0.77 (0.58-0.88)	0.753
Median LDH (IQR)	296.5 (201.25-642.25)	200 (185-250.75)	0.072
Median IL-6 (IQR)	21 (9.75-57)[3]	27 (12.25-38.91)	0.631
Median MoCA score at baseline (IQR)	27 (26.25-28)	24.5 (22-25)	<0.001
Median MMSE score at baseline (IQR)	29 (28-29)[2]	28.5 (27.25-29.75)	0.619

[1]Cognitive impairment was defined by the total MoCA score. Only 2 patients had cognitive impairment based on the MMSE test and also had cognitive impairment based on the MoCA.[2]One patient had missing data.[3]Two patients had missing data. ALL: acute lymphoblastic leukemia, AUTO: autologous hematopoietic cell transplantation, CAR-T: chimeric antigen receptor T-cell, CR: complete remission, CRP: C-reactive protein, HCT: hematopoietic cell transplantation, IL-6: interleukin-6, IQR: interquartile range, LDH: lactate

dehydrogenase, MM: multiple myeloma, MMSE: Mini-Mental State Examination, MoCA: Montreal Cognitive Assessment, NHL: non-Hodgkin lymphoma, SD: standard deviation

Supplementary Table S5. Cognitive outcomes at baseline (T1), stratified by gender, underlying diagnosis, prior HCT, and CAR-T product type.

	Male gender (N=22)	Female gender (N=14)	p-value
MoCA category, n (%)<br>Normal cognitive function<br>Cognitive impairment	 16 (72.7)<br>6 (27.3)	 8 (57.1)<br>6 (42.9)	0.334
Median MoCA score at baseline (IQR)	26.5 (25-28)	26.5 (22.75-27.25)	0.432
Median MMSE score at baseline (IQR)	29 (28-30)	29 (28-29)[1]	0.827
	NHL (N=28)	Other diagnosis (N=8)	
MoCA category, n (%)<br>Normal cognitive function<br>Cognitive impairment	 18 (64.3)<br>10 (35.7)	 6 (75)<br>2 (25)	0.691
Median MoCA score (IQR)	26 (25-27)	27 (25.25-28.75)	0.320
Median MMSE score (IQR)	29 (28-29)	29 (28-29)	0.825
	No-HCT (N=30)	HCT (N=6)	
MoCA category, n (%)<br>Normal cognitive function<br>Cognitive impairment	 20 (66.7)<br>10 (33.3)	 4 (66.7)<br>2 (33.3)	1.000
Median MoCA score (IQR)	26.5 (25-28)	26.5 (24.25-27.5)	0.918

Median MMSE score (IQR)	29 (28-29)[1]	29 (28-29.25)	0.782
	Axi-cel (N=23)	Other CAR-T cell product (N=13)	
MoCA category, n (%)<br>Normal cognitive function<br>Cognitive impairment	 15 (65.2)<br>8 (34.8)	 9 (69.2)<br>4 (30.8)	1.000
Median MoCA score (IQR)	26 (25-27)	27 (25-28.5)	0.558
Median MMSE score (IQR)	29 (28-29)[1]	29 (27.25-29)	0.548

[1]One patient had missing data. Axi-cel: axicabtagene ciloleucel, CAR-T: chimeric antigen receptor T-cell, HCT: hematopoietic cell transplantation, IQR: interquartile range, MMSE: Mini-Mental State Examination, MoCA: Montreal Cognitive Assessment, NHL: non-Hodgkin lymphoma.

Supplementary Table S6. Univariate logistic regression analysis of factors associated with cognitive impairment, assessed by the MoCA test at T1 following CAR-T cell infusion.

	OR	95% CI	p-value
Age	1.05	1.000-1.12	0.0802
Female gender	2.000	0.484-8.51	0.337
NHL	1.67	0.312-12.9	0.573
Axicabtagene ciloleucel	1.20	0.285-5.56	0.806
Previous HCT	1.000	0.123-6.09	1.000
Lines of previous treatment	1.16	0.675-1.99	0.582
Ferritin baseline (LnFerritin)	0.851	0.537-1.31	0.467
CRP baseline (LnCRP)	0.476	0.215-0.861	0.0315
PLTs baseline	1.000	0.994-1.01	0.650
LDH baseline (LnLDH)	0.314	0.0578-1.00	0.104
IL-6 baseline (LnIL-6)	1.00	0.582-1.64	0.999

CAR-T: chimeric antigen receptor T-cell, CI: confidence interval, CRP: C-reactive protein, HCT: hematopoietic cell transplantation, IL-6: interleukin-6, Ln: natural logarithmic transformation, LDH: lactate dehydrogenase, MoCA: Montreal Cognitive Assessment, NHL: non-Hodgkin lymphoma, OR: odds ratio, PLTs: platelets.

Supplementary Table S7. Multivariate logistic regression analysis models for the presence of cognitive impairment at T1 (baseline), assessed by the MoCA test. Variables with p-value < 0.1 in univariate logistic regression analysis (Supplementary Table S6) were included. Moreover, the variables included were checked for multicollinearity by calculating VIF. In all cases, VIF was less than 2.

	OR	95% CI	p-value
Age (per year)	1.033	0.977–1.105	0.284
Baseline CRP (LnCRP)	0.53	0.236–0.998	0.076

CI: confidence interval, CRP: C-reactive protein, Ln: natural logarithmic transformation, MoCA: Montreal Cognitive Assessment, OR: odds ratio, VIF: variance inflation factor.

Supplementary Table S8. Baseline and infusion characteristics, inflammatory markers, and cognitive outcomes according to cognitive status after CAR-T cell infusion (T2).

	Normal cognitive function following infusion (N=24)	Cognitive impairment following infusion (N=11)[1]	p-value
Mean age (±SD)	45.21 (±17.1)	60.64(±6.9)	<0.001
Gender, n (%)<br>Male<br>Female	 14 (58.3)<br>10 (41.7)	 7 (63.6)<br>4 (36.4)	 1.000
Disease, n (%)<br>NHL<br>ALL<br>MM	 18 (75)<br>5 (20.8)<br>1 (4.2)	 9 (81.8)<br>0<br>2 (18.2)	0.1325
Disease phase, n (%)<br>CR<br>Refractory/ active	 4 (16.7%)<br>15 (62.5%)	 1 (9.1%)<br>8 (72.7%)	1.0000

Relapsed	5 (20.8%)	2 (18.2%)	
Median lines of previous treatment (IQR)	3 (2-4)[2]	3 (2-4.25)[2]	0.603
Previous HCT, n<br>(%)<br>No<br>Auto<br>Allo	 20 (83.3)<br>1 (4.2)<br>3 (12.5)	 9 (81.8)<br>2 (18.2)<br>0	0.2229
CAR-T cell product,<br>(%)<br>Axicabtagene<br>ciloleucel<br>Brexucabtagene<br>autoleucel<br>Tisagenlecleucel<br>Ciltacabtagene<br>autoleucel<br>PHE-885	 <br>14 (58.3)<br><br>5 (20.8)<br><br>4 (16.7)<br>0<br><br>1 (4.2)	 8 (72.7)<br>1 (9.1)<br><br>0<br><br>1 (9.1)<br>1 (9.1)	0.2671
Median ferritin at baseline (IQR)	393 (105-649.725)	176 (66-260)	0.085
Median CRP at baseline (IQR)	0.92 (0.58-3.64)	0.25 (0.13-0.8)	0.004
Mean Platelets at baseline (±SD)	170.8 (±100.6)	181.18 (±56.5)	0.753
Median creatinine at baseline (IQR)	0.70 (0.55-0.96)	0.74 (0.57-0.83)	0.986
Median LDH at baseline (IQR)	246.5 (194.25-491.25)	211.0 (184-264)	0.299
Median IL-6 at baseline (IQR)	24.5 (18.5—57)	10 (8-38.64)	0.264
Mean Platelets day 0 (±SD)	127.71 (±60.6)	185.09 (±69.5)	0.018
Median creatinine day 0 (IQR)	0.61 (0.52-0.91)	0.73 (0.67-0.88)	0.174
Median LDH day 0 (IQR)	258 (171-413.5)	198 (181-264)	0.352
Cognitive function at baseline, n (%)<br>Normal<br>Impaired	 <br>19 (79.2)<br>5 (20.8)	 <br>4 (36.4)<br>7 (63.6)	0.022

Median MoCA total score at baseline (IQR)	27 (26-28)	25 (22-26)	<0.001
Median MMSE total score at baseline (IQR)	29 (29-30)	28 (28-29)	0.021
ICANS, n (%)	15 (62.5)	8 (72.7)	0.554
ICANS Grade≥II, n (%)	10 (41.7)	3 (27.3)	0.478
Median MoCA total score after infusion (IQR)	27 (26-28)	22 (19-24)	<0.001
Median MMSE total score after infusion (IQR)	29 (28-30)	26 (23-27)	<0.001

[1]Cognitive impairment was defined by the total MoCA score. Only 3 patients had cognitive impairment based on the MMSE test and had also cognitive impairment based on the MoCA.[2]One patient had missing data. ALL: acute lymphoblastic leukemia, AUTO: autologous hematopoietic cell transplantation, CAR-T: chimeric antigen receptor T-cell, CR: complete remission, CRP: C-reactive protein, HCT: hematopoietic cell transplantation, ICANS: immune effector cell–associated neurotoxicity syndrome, IL-6: interleukin-6, IQR: interquartile range, LDH: lactate dehydrogenase, MM: multiple myeloma, MMSE: Mini-Mental State Examination, MoCA: Montreal Cognitive Assessment, NHL: non-Hodgkin lymphoma, SD: standard deviation

Supplementary Table S9. Cognitive outcomes after CAR-T cell infusion (T2), stratified by gender, underlying diagnosis, prior HCT, and CAR-T product type.

	Male gender (N=21)	Female gender (N=14)	p-value
MoCA category, n (%)<br>Normal cognitive function<br>Cognitive impairment	 14 (66.7)<br>7 (33.3)	 10 (71.4)<br>4 (28.6)	1.000
Median MoCA score after infusion (IQR)	26 (24.5-28)	26 (19.75-27)	0.495

Median MMSE score after infusion (IQR)	29 (26.5-29.5)	27 (26-29)[1]	0.292
	NHL (N=27)	Other diagnosis (N=8)	
MoCA category, n (%)<br>Normal cognitive function<br>Cognitive impairment	 18 (66.7)<br>9 (33.3)	 6 (75)<br>2 (25)	1.000
Median MoCA score after infusion (IQR)	27 (24-27)	26 (23.75-28.25)	0.893
Median MMSE score after infusion (IQR)	28 (26-29)	28 (28-29)[1]	0.504
	No-HCT (N=29)	HCT (N=6)	
MoCA category, n (%)<br>Normal cognitive function<br>Cognitive impairment	 20 (69)<br>9 (31)	 4 (66.7)<br>2 (33.3)	1.000
Median MoCA score after infusion (IQR)	26 (24-27)	27 (22.5-29)	0.564
Median MMSE score after infusion (IQR)	28.5 (26-29.75)[1]	28 (26-29)	0.644
	Axi-cel (N=22)	Other CAR-T cell product (N=13)	
MoCA category, n (%)<br>Normal cognitive function<br>Cognitive impairment	 14 (63.6)<br>8 (36.4)	 10 (76.9)<br>3 (23.1)	0.478
Median MoCA score (IQR)	26 (24-27)	26 (24.5-28)	0.468
Median MMSE score (IQR)	27.5 (26-29.25)	28.5 (27.25-29)[1]	0.631

[1]One patient had missing data. CAR-T: chimeric antigen receptor T cell, HCT: hematopoietic cell transplantation, IQR: interquartile range, MMSE: Mini-Mental State Examination, MOCA: Montreal Cognitive Assessment, NHL: non-Hodgkin lymphoma

Supplementary Table S10. Univariate logistic regression analysis of factors associated with cognitive impairment, assessed by the MoCA test at T2 following CAR-T cell infusion.

	OR	95% CI	p-value
Age (per year)	1.1	1.03-1.2	0.0211
Female gender	0.8	0.171-3.43	0.766
NHL	1.5	0.277-11.7	0.657
Axicabtagene ciloleucel	1.90	0.426-10.4	0.417
Previous HCT	1.11	0.136-6.86	0.912
Lines of previous treatment	1.2	0.695-2.09	0.498
Ferritin baseline (LnFerritin)	0.784	0.474-1.24	0.31
CRP baseline (LnCRP)	0.342	0.123-0.702	0.0136
PLTs baseline	1.00	0.993-1.01	0.745
LDH baseline (LnLDH)	0.432	0.0883-1.26	0.206
IL-6 baseline (LnIL-6)	0.771	0.39-1.31	0.38
Platelets day 0	1.02	1-1.03	0.0326
LDH day 0 (LnLDH0)	0.43	0.079-1.45	0.243
Cognitive impairment at baseline[1]	6.65	1.45-35.6	0.0184
MoCA total score at baseline	0.479	0.249-0.746	0.00624
MMSE total score at baseline	0.720	0.420-1.07	0.144
ICANS of every grade	1.6	0.354-8.76	0.556
ICANS grade≥II	0.525	0.096-2.35	0.417

[1]Assesed by the MoCA test at T1. CI: confidence interval, CRP: C-reactive protein, HCT: hematopoietic cell transplantation, ICANS: immune effector cell–associated neurotoxicity syndrome, IL-6: interleukin-6, LDH: lactate dehydrogenase, LnCRP: natural logarithm of C-reactive protein, LnFerritin: natural logarithm of ferritin, LnIL-6: natural logarithm of interleukin-6, LnLDH: natural logarithm of lactate dehydrogenase, LnLDH0: natural logarithm of lactate dehydrogenase at day 0, MMSE: Mini-Mental State Examination, MoCA: Montreal Cognitive Assessment, NHL: non-Hodgkin lymphoma, OR: odds ratio, PLTS: platelets

Supplementary Table S11. Multivariate logistic regression analysis models for the presence of cognitive impairment at T2, assessed by the MoCA test. Variables with *p*-value < 0.1 in univariate logistic regression analysis (Supplementary Table S10) were included. Moreover, the variables included were checked for multicollinearity by calculating VIF. In all cases, VIF was less than 2.

	OR	95% CI	p-value
Model 1			
Age (per year)	1.09	1.003–1.236	0.093
Baseline cognitive impairment[1]	3.507	0.549–26.338	0.191
CRP at baseline (LnCRP)	0.425	0.144–0.98	0.072
Model 2			
Age (per year)	1.119	1.027–1.27	0.034
Baseline cognitive impairment[1]	5.141	0.717–52.221	0.117
Platelets at day 0	1.02	1.003–1.043	0.047

[1]Assesed by the MoCA test at T1. CI: confidence interval, CRP: C-reactive protein, LnCRP: natural logarithm of C-reactive protein, MoCA: Montreal Cognitive Assessment, OR: odds ratio, VIF: variance inflation factor.

Supplementary Table S12. Baseline and infusion characteristics, inflammatory markers, treatment-related toxicities, and cognitive outcomes according to cognitive status at 3 months following CAR-T cell infusion (T3).

	Normal cognitive	Cognitive impairment	p-value

	function<br>following 3<br>months post-<br>infusion (N=25)	following 3<br>months post-<br>infusion (N=9)[1]	
Median age (IQR)	52 (39.5-65.5)	57 (48-62.5)	0.645
Gender, n (%)<br>Male<br>Female	 17 (68)<br>8 (32)	 3 (33.3)<br>6 (66.7)	 0.116
Disease, n (%)<br>NHL<br>ALL<br>MM	 18 (72)<br>4 (16)<br>3 (12)	 8 (88.9)<br>1 (11.1)<br>0	0.8119
Disease phase, n (%)<br>CR<br>Refractory/ active<br>Relapsed	 5 (20)<br>15 (60)<br>5 (20)	 0<br>8 (88.9)<br>1 (11.1)	0.3695
Median lines of previous treatment (IQR)	3 (2-3)[2]	3 (2-4.5)[2]	0.409
Previous HCT, n (%)<br>No<br>Auto<br>Allo	 21 (84)<br>2 (8)<br>2 (8)	 7 (77.8)<br>1 (11.1)<br>1 (11.1)	1.000
CAR-T cell product, (%)<br>Axicabtagene ciloleucel<br>Brexucabtagene autoleucel<br>Tisagenlecleucel<br>Ciltacabtagene<br>autoleucel<br>PHE-885	 15 (60)<br>3 (12)<br>4 (16)<br>1 (4)<br>2 (8)	 6 (66.7)<br>3 (33.3)<br>0<br>0<br>0	0.4951
Median ferritin at baseline (IQR)	336 (122-490)	176 (44-1308.5)	0.673
Median CRP at baseline (IQR)	0.8 (0.41-1.64)	0.4 (0.14-1.89)	0.280
Mean platelets at baseline (±SD)	168 (±85.676)	176 (±72.235)	0.978
Median creatinine at baseline (IQR)	0.81 (0.6-1)	0.61 (0.52-0.79)	0.079

Median LDH at baseline (IQR)	236 (194.5-421.5)	205 (183.5-393)	0.539
Median IL-6 at baseline (IQR)	20 (10-39)	30 (21.5-85)	0.145
Mean Platelets day 0 (±SD)	138.8 (±61.39)	157.89 (±86.98)	0.480
Median creatinine day 0 (IQR)	0.74 (0.58-0.99)	0.67 (0.49-0.71)	0.094
Median LDH day 0 (IQR)	234 (177-355.5)	183 (172.5-385)	0.489
Median LDH day 14 (IQR)	213 (161.5-288.5)	227 (163.5-360)	0.701
Median creatinine day 14 (IQR)	0.79 (.59-1.03)	0.60 (0.52-0.73)	0.086
Median platelets day 14 (IQR)	174 (28-234.5)	83 (34.5-154)	0.316
Median CRP day 14 (IQR)	0.15 (0.05-0.47)	0.06 (0.04-0.19)	0.151
Median max IL-6 post-infusion (IQR)	721.5 (106.5-4514.7)	1454.5 (457.5-3207.5)	0.592
Median max CRP post-infusion (IQR)	6.13 (2.35-12.21)	2.39 (1.72-3.9)	0.027
Cognitive function at baseline, n (%)<br>Normal<br>Impaired	 <br>20 (80)<br>5 (20)	 <br>2 (22.2)<br>7 (77.8)	0.04
Median MoCA total score at baseline (IQR)	27 (26-28)	23 (21.5-25.5)	0.02
Median MMSE total score at baseline (IQR)	29 (28-29.75)	28 (26-29)	0.166
ICANS, n (%)	17 (68)	6 (66.7)	1.000
ICANS Grade≥II, n (%)	11 (44)	2 (22.2)	0.427
CRS, n (%)	22 (88)	9 (100)	0.549
Grade≥ II CRS, n (%)	18 (72)	7 (77.8)	1.000
Tocilizumab, n (%)	22 (88)	9 (100)	0.549
Corticosteroids, (%)	14 (56)	7 (77.8)	0.427
Median MoCA total score after infusion (IQR)	26 (25.5-27.5)	25 (18.5-27)	0.086

Median MMSE total score after infusion (IQR)	29 (27-30)	26 (22-27.5)	0.002
Cognitive function after infusion, n (%)<br>Normal<br>Impaired	 <br>19 (76)<br>6 (24)	 <br>4 (44.4)<br>5 (55.6)	0.111
Median MoCA total score at 3 months post-infusion (IQR)	27 (26-28)	23 (20.5-24)	<0.001
Median MMSE total score at 3 months post-infusion (IQR)	29 (28-29)	27 (26-28.5)	0.018

[1]Cognitive impairment was defined by the total MoCA score. Only 1 patient had cognitive impairment based on the MMSE test and had also cognitive impairment based on the MoCA.[2]One patient had missing data. ALL: acute lymphoblastic leukemia, AUTO: autologous, ALLO: allogeneic, CAR-T: chimeric antigen receptor T- cell, CR: complete remission, CRP: C-reactive protein, CRS: cytokine release syndrome, HCT: hematopoietic cell transplantation, ICANS: immune effector cell–associated neurotoxicity syndrome, IL-6: interleukin-6, IQR: interquartile range, LDH: lactate dehydrogenase, MM: multiple myeloma, MMSE: Mini-Mental State Examination, MoCA: Montreal Cognitive Assessment, NHL: non-Hodgkin lymphoma, SD: standard deviation.

Supplementary Table S13. Cognitive outcomes at 3 months following CAR-T cell infusion (T3), stratified by gender, underlying diagnosis, prior HCT, and CAR-T product type.

	Male gender (N=20)	Female gender (N=14)	p-value
MoCA category, n (%)<br>Normal cognitive function<br>Cognitive impairment	 17 (85)<br>3 (15)	 8 (57.1)<br>6 (42.9)	0.116
Median MoCA score at 3 months post-infusion (IQR)	26 (25-27.75)	25.5 (23-28)	0.592

Median MMSE score at 3 months post-infusion (IQR)	29 (27-29)	29[1] (27-29)	0.899
	NHL (N=26)	Other diagnosis (N=8)	
MoCA category, n (%)<br>Normal cognitive function<br>Cognitive impairment	 18 (69.2)<br>8 (30.8)	 7 (87.5)<br>1 (12.5)	0.403
Median MoCA score at 3 months post-infusion (IQR)	26.5 (25-28)	26 (23.75-28)	0.563
Median MMSE score at 3 months post-infusion (IQR)	29 (27-30)	29[1] (27-29)	0.232
	No-HCT (N=28)	HCT (N=6)	
MoCA category, n (%)<br>Normal cognitive function<br>Cognitive impairment	 21 (75)<br>7 (25)	 4 (66.7)<br>2 (33.3)	0.644
Median MoCA score at 3 months post-infusion (IQR)	26 (24.25-28)	25.5 (23-28)	0.644
Median MMSE score at 3 months post-infusion (IQR)	29[1] (27-29)	29 (28.5-29.25)	0.205
	Axi-cel (N=21)	Other CAR-T cell product (N=13)	
MoCA category, n (%)<br>Normal cognitive function<br>Cognitive impairment	 15 (71.4)<br>6 (28.6)	 10 (76.9)<br>3 (23.1)	1.000
Median MoCA score at 3 months post-infusion (IQR)	26 (24-28)	26 (24.5-28)	0.944

Median MMSE score at 3 months post-infusion (IQR)	29 (27-29)	28.5[1] (27.25-29)	0.897

[1]One patient had missing data. CAR-T: chimeric antigen receptor T cell, HCT: hematopoietic cell transplantation, IQR: interquartile range, MMSE: Mini-Mental State Examination, MoCA: Montreal Cognitive Assessment, NHL: non-Hodgkin lymphoma,

Supplementary Table S14. Comparison of cognitive impairment prevalence, median MoCA, and median MMSE total score between patients with ICANS and those without at T1, T2, T3, and T4.

	ICANS	Non-ICANS	p-value
Cognitive impairment at baseline (T1), n (%)			0.720
Normal	16 (69.6)	8 (61.5)	
Impaired	7 (30.4)	5 (38.5)	
Median MoCA total score at baseline (T1) (IQR)	27 (25-27)	26 (25-28)	0.697
Median MMSE total score at baseline (T1) (IQR)	28.5 (28-29)	29 (29-30)	0.121
Cognitive impairment after infusion (T2), n (%)			0.709
Normal	15 (65.2)	9 (75)	
Impaired	8 (34.8)	3 (25)	
Median MoCA total score after infusion (T2) (IQR)	26 (24-27)	27 (24.5-28)	0.278

Median MMSE total score after infusion (T2) (IQR)	27.5 (26-29.25)	29 (26.5-29)	0.488
Cognitive impairment at 3 months (T3), n (%)<br>Normal<br>Impaired	 <br>17 (73.9)<br>6 (26.1)	 <br>8 (72.7)<br>3 (27.3)	1.000
Median MoCA total score at 3 months (T3) (IQR)	26 (24-28)	26 (22-28)	0.690
Median MMSE total score at 3 months (T3) (IQR)	29 (27-29)	29 (26-29)	0.336
Cognitive impairment at 6 months (T4), n (%)<br>Normal<br>Impaired	 <br>18 (81.8)<br>4 (18.2)	 <br>9 (81.8)<br>2 (18.2)	1.000
Median MoCA total score at 6 months (T4) (IQR)	25.5 (25-27.25)	27 (25-27)	0.895
Median MMSE total score at 6 months (T4) (IQR)	28 (27-29)	29 (28-29)	0.317

ICANS: immune effector cell–associated neurotoxicity syndrome, IQR: interquartile range, MMSE: Mini-Mental State Examination, MoCA: Montreal Cognitive Assessment.

Supplementary Table S15. Comparison of cognitive impairment prevalence, median MoCA, and median MMSE total score between patients with ICANS ≥ II and the rest of the study participants at T1, T2, T3, and T4.

	ICANS grade≥II	ICANS I or non-ICANS	p-value
Cognitive impairment at baseline (T1), n (%)<br>Normal<br>Impaired	 10 (76.9)<br>3 (23.1)	 14 (60.9)<br>9 (39.1)	0.468
Median MoCA total score at baseline (T1) (IQR)	27 (25.5-28)	26 (25-28)	0.296
Median MMSE total score at baseline (T1) (IQR)	28.5 (28-29.75)	29 (28-29)	0.771
Cognitive impairment after infusion (T2), n (%)<br>Normal<br>Impaired	 10 (76.9)<br>3 (23.1)	 14 (63.6)<br>8 (36.4)	0.478
Median MoCA total score after infusion (T2) (IQR)	26 (25.5-27)	26 (23.5-28)	0.880
Median MMSE total score after infusion (T2) (IQR)	29 (26.25-30)	28 (26-29)	0.292
Cognitive impairment			0.427

at 3 months (T3), n (%)<br>Normal<br>Impaired	 11 (84.6)<br>2 (15.4)	 14 (66.7)<br>7 (33.3)	
Median MoCA total score at 3 months (T3) (IQR)	26 (25-28)	26 (23.5-28)	0.309
Median MMSE total score at 3 months (T3) (IQR)	29 (27.25-29)	29 (27-29)	0.567
Cognitive impairment at 6 months (T4), n (%)<br>Normal<br>Impaired	 10 (83.3)<br>2 (16.7)	 17 (81.0)<br>4 (19.0)	1.000
Median MoCA total score at 6 months after infusion (T4) (IQR)	26 (25-27.75)	26 (25-27)	0.671
Median MMSE total score at 6 months after infusion (T4) (IQR)	28 (27.25-28)	29 (28-29)	0.104

ICANS: immune effector cell–associated neurotoxicity syndrome, IQR: interquartile range, MMSE: Mini-Mental State Examination, MoCA: Montreal Cognitive Assessment.

Supplementary Table S16. Comparison of cognitive impairment prevalence, median MoCA, and median MMSE total score between patients with CRS ≥ II and the rest of the study participants at T1, T2, T3, and T4.

	CRS grade≥II	CRS I or non-CRS	p-value
Cognitive impairment at baseline (T1), n (%)			0.126
Normal	20 (74.1)	4 (44.4)	
Impaired	7 (25.9)	5 (55.6)	
Median MoCA total score at baseline (T1) (IQR)	27 (25-28)	25 (24-27)	0.067
Median MMSE total score at baseline (T1) (IQR)	27 (28-29)	29 (29-29.5)	0.288
Cognitive impairment after infusion (T2), n (%)			0.416
Normal	19 (73.1)	5 (55.6)	
Impaired	7 (26.9)	4 (44.4)	
Median MoCA total score after infusion (T2) (IQR)	26 (25-27)	26 (19-28.5)	0.697
Median MMSE total score after infusion (T2) (IQR)	28 (27-29.5)	28 (25.5-29)	0.442
Cognitive impairment at 3 months (T3), n (%)			1.000

Normal<br>Impaired	18 (72)<br>7 (28)	7 (77.8)<br>2 (22.2)	
Median MoCA total score at 3 months (T3) (IQR)	26 (24-28)	27 (23.5-28)	0.878
Median MMSE total score at 3 months (T3) (IQR)	29 (27-29)	29 (25.5-29)	0.953
Cognitive impairment at 6 months (T4), n (%)<br>Normal<br>Impaired	 <br><br><br>20 (83.3)<br>4 (16.7)	 <br><br><br>7 (77.8)<br>2 (22.2)	1.000
Median MoCA total score at 6 months after infusion (T4) (IQR)	26 (25-27)	27 (22.5-27)	0.953
Median MMSE total score at 6 months after infusion (T4) (IQR)	28 (28-29)	28 (28-29)	0.858

CRS: cytokine release syndrome, IQR: interquartile range, MMSE: Mini-Mental State Examination, MoCA: Montreal Cognitive Assessment.

Supplementary Table S17. Comparison of cognitive impairment prevalence, median MoCA, and median MMSE total score between patients with ICANS grade III–IV and the rest of the study participants at T1, T2, T3, and T4.

	ICANS grade III or IV	ICANS grade I, II, or no ICANS	p-value
Cognitive impairment at baseline (T1), n (%)<br>Normal<br>Impaired	 <br>3 (50)<br>3 (50)	 <br>21 (70)<br>9 (30)	0.378
Median MoCA total score at baseline (T1) (IQR)	26 (24.25-27.5)	26.5 (25-28)	0.725
Median MMSE total score at baseline (T1) (IQR)	28.5 (28-29)	29 (27.75-30)	0.915
Cognitive impairment after infusion (T2), n (%)<br>Normal<br>Impaired	 <br>4 (66.7)<br>2 (33.3)	 <br>20 (69)<br>9 (31)	1.000
Median MoCA total score after infusion (T2) (IQR)	26 (22.5-27)	26 (24-28)	0.428
Median MMSE total score after infusion (T2) (IQR)	28 (23.75-30)	28 (26.25-29)	0.982
Cognitive impairment			0.644

at 3 months (T3), n (%)<br>Normal<br>Impaired	 <br>4 (66.7)<br>2 (33.3)	 <br>21 (75)<br>7 (25)	
Median MoCA total score at 3 months (T3) (IQR)	25.5 (23-28.25)	26 (24.25-28)	0.843
Median MMSE total score at 3 months (T3) (IQR)	28 (26.75-29.25)	29 (27-29)	0.768
Cognitive impairment at 6 months (T4), n (%)<br>Normal<br>Impaired	 <br><br><br>4 (66.7)<br>2 (33.3)	 <br><br><br>23 (85.2)<br>4 (14.8)	0.295
Median MoCA total score at 6 months after infusion (T4) (IQR)	25 (24.75-28.25)	26 (25-27)	0.838
Median MMSE total score at 6 months after infusion (T4) (IQR)	28 (27.25-28)	29 (28-29)	0.132

ICANS: immune effector cell-associated neurotoxicity, IQR: interquartile range, MMSE: Mini-Mental State Examination, MoCA: Montreal Cognitive Assessment.

Supplementary Table S18. Cognitive outcomes in patients with CRS grade III–IV.

Patient No	CRS grade	ICANS grade	MoCA cognitive status and total	MoCA cognitive status and total	MoCA cognitive status and total	MoCA cognitive status and total

			score at baseline	score after infusion	score at 3 months	score at 6 months
1	III	III	Normal (29)	Normal (26)	Normal (28)	Normal (29)
2	IV	IV	Normal (27)	Normal (26)	Normal (29)	Normal (28)
3	IV	No	Normal (26)	NA	NA	NA

CRS: cytokine release syndrome, ICANS: immune effector cell-associated neurotoxicity, MoCA: Montreal Cognitive Assessment, NA: not available
